# Cyberbullying Among Traditional and Complementary Medicine Practitioners in the Workplace: Protocol for a Cross-sectional Descriptive Study

**DOI:** 10.2196/29582

**Published:** 2021-08-12

**Authors:** Yun Jin Kim, Linchao Qian, Muhammad Shahzad Aslam

**Affiliations:** 1 School of Traditional Chinese Medicine Xiamen University Malaysia Sepang Malaysia

**Keywords:** cyberbullying, traditional medicine, workplace, practitioners, medical professional

## Abstract

**Background:**

Cyberbullying is becoming prevalent among health care professionals and may cause a variety of mental health issues. Traditional and complementary medicine practitioners remain an important pillar of the health care system in Malaysia.

**Objective:**

This paper presents a study protocol for an online survey (Cyberbullying Among Traditional and Complementary Medicine Practitioner [TCMPs]) that will collect the first nationwide representative data on cyberbullying behavior among traditional and complementary medicine practitioners in Malaysia. The objectives of the survey are to (1) evaluate the cyberbullying behavior among traditional and complementary medicine practitioners in Malaysia, (2) identify sociodemographic and social factors related to cyberbullying, and (3) evaluate the association between cyberbullying behavior, sociodemographic, and social factors.

**Methods:**

A snowball sampling strategy will be applied. Traditional and complementary medicine practitioners who are permanent Malaysian residents will be randomly selected and invited to participate in the survey (N=1023). Cyberbullying behavior will be measured using the Cyberbullying Behavior Questionnaire (CBQ). Data on the following items will be collected: work-related bullying, person-related bullying, aggressively worded messages, distortion of messages, sending offensive photos/videos, hacking computers or sending a virus or rude message, and threatening messages about personal life or family members. We will also collect data on participants’ sociodemographic characteristics, social factors, and substance abuse behavior.

**Results:**

This cross-sectional descriptive study was registered with Research Registry (Unique Identifying Number 6216; November 05, 2020). This research work (substudy) is planned under a phase 1 study approved by the Research Management Centre, Xiamen University Malaysia. This substudy has been approved by the Research Ethics Committee of Xiamen University Malaysia (REC-2011.01). The cross-sectional survey will be conducted from July 01, 2021, to June 30, 2022. Data preparation and statistical analyses are planned from January 2022 onward.

**Conclusions:**

The current research can contribute to identify the prevalence of workplace cyberbullying among Malaysian traditional and complementary medicine practitioners. The results will help government stakeholders, health professionals, and education professionals to understand the psychological well-being of Malaysian traditional and complementary medicine practitioners.

**Trial Registration:**

Research Registry Unique Identifying Number 6216; https://tinyurl.com/3rsmxs7u

**International Registered Report Identifier (IRRID):**

PRR1-10.2196/29582

## Introduction

### Background

Adverse consequences of cyberbullying behavior in the workplace are well-documented [1,2]. The negative effects of cyberbullying behavior are harmful or aggressive communications [3], expressing negative emotions [4], and e-harassment [5]. Cyberbullying is a severe threat to the workplace that results in job dissatisfaction, mental strain [6], and perceived organizational injustice, which in turn increases the perceived job stress that eventually results in cyberbullying [3]. Cyberbullying is defined as a repetitive negative (harmful) behavior by a person (perpetrator) to intentionally hurt an individual (affected party) through technological means, such as SMS text messages or email. In most cases, this involves an imbalance of power between the perpetrator (usually anonymous) and the affected individual. The perpetrator’s action is generally considered more severe in the public domain than in the private domain [7].

The prevalence of cyberbullying in the workplace has raised some serious global public health concern. A Swedish survey estimated the prevalence of workplace cyberbullying to be 9.7% [8], based on Leymann’s cut-off criterion [9]. Gardner et al [10] performed a study on predictors of workplace bullying and cyberbullying in New Zealand, and found that among the total study participants (N=826), 15% (n=123) experienced bullying and 2.8% (n=23) experienced cyberbullying (2.8%) within the last 6 months. Workplace bullying in different countries among different staff members have been reported in many studies; for example, the prevalence of bullying among hospital employees in Austria was reported to be 26.6% [11], among university employees in Finland to be 16.9% [12], and among health and welfare managers in Norway to be 8.6% [13]. Bullying in workplace is also reported from studies conducted in Ireland (16.9%) [14] and Portugal (33.5%) [15,16]. By contrast, there is very limited research on workplace cyberbullying.

Cyberbullying behavior has psychological effects on the affected individual, such as social anxiety [17], emotional distress [18], and depression [19]. However, the exact biological mechanism underlying cyberbullying remains unknown. Cabrera et al [20] reported on the role of cortisol in cyberbullying behavior, with the level of this hormone being higher among affected individuals due to increased activity of the hypothalamic–pituitary–adrenocortical axis, which plays an important in the management of stress.

The World Health Organization has expressed concern with the prevalence of bullying among students and employees globally [21-23]. Recently, The United Nations Educational, Scientific, and Cultural Organization and the Government of Ireland, Dublin City University, have developed a partnership to increase institutional capacities on cyberbullying awareness through knowledge sharing and collaborative work [24]. The International Labour Organization sets benchmarks for defining, preventing, and responding to violence at the workplace and recognizes bullying under “aggressive behavior” [25].

### Objectives

Using the workplace Cyberbullying Behavior Questionnaire (CBQ), this study aims to provide the first nationally representative data on cyberbullying behavior among traditional and complementary medicine practitioners in Malaysia. Objectives of the CBQ survey are to (1) evaluate the cyberbullying behavior among traditional and complementary medicine practitioners in Malaysia, (2) identify sociodemographic and social factors related to cyberbullying, and (3) evaluate the association between cyberbullying behavior, sociodemographic, and social factors. In the following sections, we discuss the research design and methods and present an overview of methodological challenges, strengths, and limitations related to the study design and sampling strategy.

## Methods

### Study Design

This is a cross-sectional descriptive study performed using the Cyberbullying Behavior Questionnaire (CBQ and its short version [CBQ-S]), which was administered to traditional and complementary medicine practitioners in Malaysia. The questionnaire includes 32 questions (see Multimedia Appendix 1) in a closed-ended question format. The standardized checklist for the Strengthening the Reporting of Observational Studies in Epidemiology (STROBE) recommendations was used to ensure that all the elements recommended were addressed within this section to participate in the study, including a link to the platform where all the information related to the project, its objectives, and expected outcomes can be found [26]. Traditional and complementary medicine practitioners will receive an online invitation using SurveyMonkey to participate in the study, including a link to the platform where all the information related to the project, its objectives, and expected outcomes can be found [27]. The online survey will follow the CHERRIES guideline [28] to maintain the quality of the web-based survey. Malaysia is a highly digitally networked nation, with nearly 90% of households using the internet, mostly through mobile broadband plans on smartphones. The rationale to conduct an online survey is the ease of internet availability in Malaysia [29].

### Study Population

Individuals were selected for study participation according to the following inclusion criteria: traditional and complementary medicine practitioners working in the public and private sector in Malaysia; and currently practicing in any one of the practice areas recognized by the Ministry of Health Malaysia (Traditional Malay Medicine, Traditional Chinese Medicine, Traditional Indian Medicine, homeopathy, chiropractic, osteopathy, and Traditional Islamic Medicine).

All eligible participants will be contacted through official Facebook pages of the Malaysian Society for Complementary Medicine, Federation of Chinese Physicians and Acupuncturists Associations Malaysia, Malaysian Chinese Medical Association, TCM & Western Naturopathic Malaysia, Traditional Malay Medicine Association, Majlis Perubatan Homeopathy Malaysia, Association of Chiropractic Malaysia, Malaysia Osteopathy Association, and Malaysian Health Qigong Association.

Individuals were excluded from study participation according to the following exclusion criteria: not traditional and complementary medicine practitioners in one of the recognized practice areas and practitioners who did not give consent.

### Sampling Strategies

**Figure 1:**
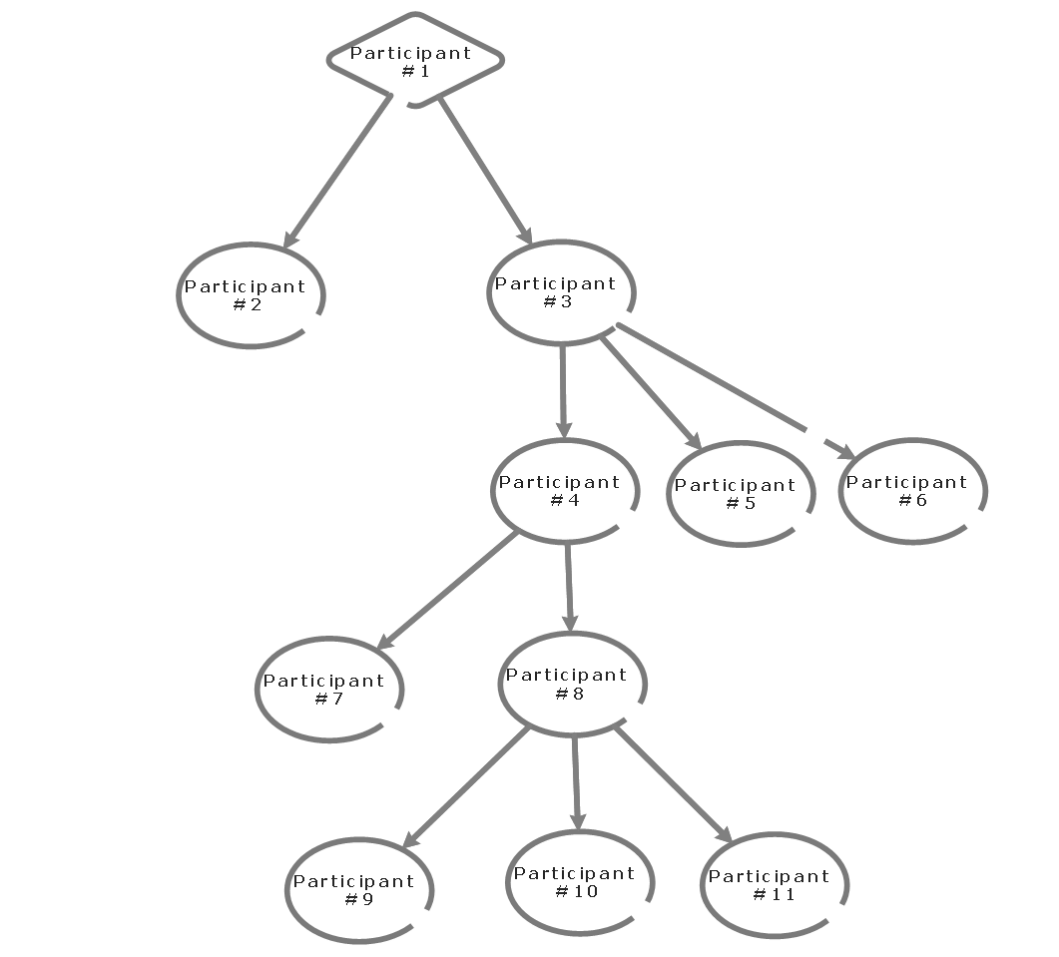
Overview of the snowball sampling strategy.

Using traditional sampling strategies to recruit hard-to-reach population faces several hurdles. In this regard, a snowball sampling method is valuable. It is a technique to find research participants with the help of name suggestions from a single participant: initially, 1 participant is identified and then a chain of participants related to the first one is identified. This remains one of the valuable sampling strategies in descriptive studies [30]. We will use snowball sampling on the Facebook pages of official traditional and complementary medicine practitioner associations (Figure 1). A list of well-known associations is mentioned above (see inclusion criteria).

### Sample Size

Traditional and complementary medicine practitioners will be selected from both public and private sectors in Malaysia. The total number of traditional and complementary medicine practitioners in Malaysia is approximately 15,000 (data as of 2011) [31]. However, no data are available on how many practitioners are serving in the general and urban population. Because of the COVID-19 pandemic, we cannot directly contact the practitioners. Therefore, we will conduct a nonprobability snowball sampling involving 1023 participants (3% precision and 95% confidence level).

### Data Protection and Ethical Approval

All data are stored in a password-protected electronic format on OneDrive cloud (Microsoft). To ensure participant anonymity, the survey will not contain any information that will personally identify the participants. The study results will be used for research purposes only. This study will be conducted according to the Declaration of Helsinki and the guidelines of the National Committee for Clinical Research [32]. The study has been approved by the Research Ethics Committee of Xiamen University Malaysia (REC-2011.01). Data collection is expected to happen between July 01, 2021, and June 30, 2022. Duration of the online survey is 15-20 minutes. All participants will be given a study information sheet. An electronic version of the informed consent form will be made available within the survey (SurveyMonkey [27]).

### Data Collection and Data Handling

**Figure 2:**
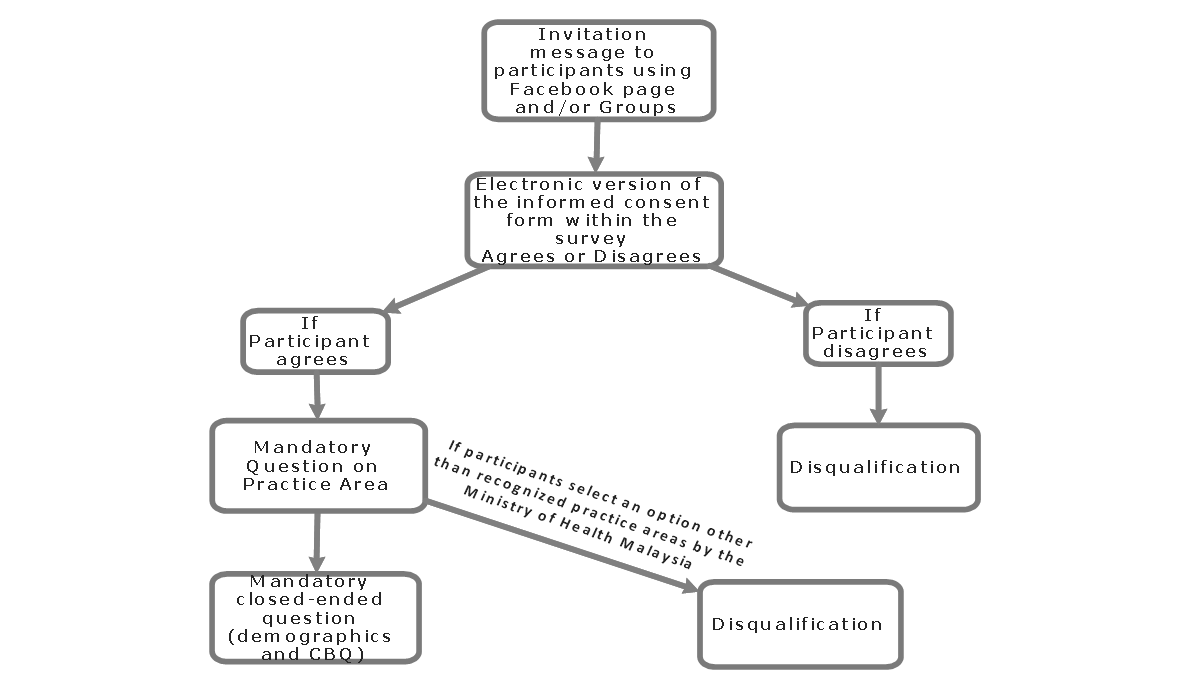
Overview of the study flow and informed consent procedure.

Participants will use SurveyMonkey to accept or decline participation. Participants will be invited via Facebook pages of medical associations. There will be a reminder every 2 weeks to follow-up on the status of the questionnaire with participants. Figure 2 presents an overview of the study flow and informed consent procedure.

### Measurements

The items in the questionnaire were chosen according to previous studies by Einarsen et al [33], Farley [34], and Forssell [8,35] and aimed for a comprehensive assessment of all aspects of workplace cyberbullying among traditional and complementary medicine practitioners in Malaysia (Table 1). There will be no public involvement in the design of this study. The results will be presented in scientific meetings worldwide and published in peer-reviewed open-access journals to disseminate the outcomes. The Cronbach α values for CBQ and CBQ-S in the study by Forssell et al [35] were .76 (Swedish sample) and .95 (American sample). The Negative Acts Questionnaire-Revised (NAQ-R) has a Cronbach α value of .90. Therefore CBQ, CBQ-S, and NAQ-R are reliable and valid instruments for the evaluation of workplace cyberbullying [8,33].

**Table 1:** Overview of the topics and measures applied in the Cyberbullying Behavior Questionnaire (N=31 items).

Topic/parameter and measure		Source	Number of items	Assessing objectives^a^
**Sociodemographics**				
	Sex, age group, and current relationship status	N/A^b^	3	2
**Education**				
	Level of education	N/A	1	2
**Socioeconomic status**				
	Household income	N/A	1	2
**Health status**				
	Substance abuse	Pattern of Substance and Drug Misuse Among Youth in Malaysia [36]	1	2
**Geography**				
	Working sector, location (city)	N/A	2	2
**Working status**				
	Community participant work, current job level, practice area	Official Portal of Traditional and Complementary Medicine Division [37]	3	2
**Cyberbullying Behavior Questionnaire**				
	Work-related bullying, person-related bullying	Negative Acts Questionnaire-Revised [33]	11	1 and 3
	Aggressively worded messages, distortion of messages	The Measurement and Impact of Workplace Cyberbullying [34]	2	1 and 3
	Posted offensive photos/videos, hacking computer or sending virus or rude message, attaching or threatening messages about personal life or family	Forssell Cyberbullying Behavior Questionnaire [8]	7	1 and 3

^a^Objective 1: To evaluate the cyberbullying behavior among traditional and complementary medicine practitioners in Malaysia; objective 2: To identify sociodemographic and social factors related to cyberbullying; and objective 3: To evaluate the association between cyberbullying behavior, sociodemographic, and social factors.

^b^N/A: not applicable.

### Data Management, Data Preparation, and Data Analysis

Descriptive statistics and exploratory structural equation modeling will be used to assess sociodemographic and social factors related to cyberbullying and evaluate the association between cyberbullying behavior, sociodemographic, and social factors. Statistical analysis will be performed using SPSS (version 26) and SPSS AMOS/ADANCO (IBM).

## Results

This cross-sectional descriptive study was registered with Research Registry (Unique Identifying Number 6216; November 05, 2020). This research work (substudy) is planned under a phase 1 study approved by the Research Management Centre, Xiamen University Malaysia, whose protocol has already been published [38]. This substudy has been approved by the Research Ethics Committee of Xiamen University Malaysia (REC-2011.01). Data preparation and statistical analyses are planned from January 2022 onward.

## Discussion

This exploratory study will provide the first nationally representative data on workplace cyberbullying for traditional and complementary medicine practitioners in Malaysia. The data collected and analyzed will explore the relationship between workplace cyberbullying and social factors. A significant strength of the study is the use of a validated measurement tool (CBQ and CBQ-S), which is a combination of different instruments validated for different population groups (Swedish and American) [35]. Compared with the Copenhagen Psychosocial Questionnaire (COPSOQ III) [39], which encompasses a broad range of psychosocial aspects of modern work life, the CBQ possesses an excellent Cronbach α value of .95 when applied in an American population.

CBQ and CBQ-S are specifically administered to evaluate cyberbullying behavior in the workplace. Understanding the prevalence of cyberbullying is the first step in the formulation of evidence-based interventions for promoting the mental health of both perpetrator(s) and affected individual(s). Our sampling strategy has both strengths and limitations. Participants will be conveniently selected from the Facebook pages of various professional associations of traditional and complementary medicine practitioners using the snowball sampling method [40]. The survey will also follow CHERRIES guidelines [28] for the design, obtaining of informed consent, development, survey administration, response rates evaluation, and analysis. Because of time constraints, it is not feasible to perform a nonresponder bias survey, which could help identify the factors associated with the lack of response [41,42].

The CBQ survey results will provide data to identify the prevalence of workplace cyberbullying among traditional and complementary medicine practitioners, identify a correlation between social factors and cyberbullying behavior, and guide the implementation of related interventions for traditional and complementary medicine practitioners in a Malaysian context. Data from this survey will help improve mental health strategies to promote mental health education among health care professionals.

## Appendix

Multimedia Appendix 1 The Cyberbullying Behavior Questionnaire.
