# Supplementary material for: Cyberbullying Among Traditional and Complementary Medicine Practitioners in the Workplace: Protocol for a Cross-sectional Descriptive Study
Source: JMIR Res Protoc. 2021 Aug 12;10(8):e29582. doi: 10.2196/29582 (PMC8391741; doi:10.2196/29582)
Supplement: Multimedia Appendix 1 [file resprot_v10i8e29582_app1.pdf]

Cyber-bullying among Traditional and Complementary Medicine Practitioners at workplace cross-sectional descriptive study

Workplace Cyber-bullying

<u>Title of Research: Cyber-bullying among Traditional and Complementary Medicine Practitioners at workplace: A cross-sectional descriptive study</u>

Principal Investigator: Dr. Muhammad Shahzad Aslam
Co-Investigator: Dr. Yun Jin Kim; Dr. Linchao Qian
Funding: Research Management Center, Xiamen University Malaysia (XMUMRF/2020-C6/ITCM/0005)
What is the objective of Study?
The purpose of this survey is to investigate workplace cyber-bullying on Traditional and Complementary Medicine Practitioners in Malaysia. This is a research project being conducted by Dr. Muhammad Shahzad Aslam, School of Traditional Chinese Medicine at Xiamen University Malaysia.

Who Can take part in this study?
Traditional and Complementary Medicine Practitioners (TCMPs) working in public sector, private sector in Malaysia. The participants from all recognized practice areas by Ministry of health Malaysia (Traditional Malay Medicine, Traditional China Medicine, Traditional India Medicine, Homeopathy, Chiropractic, Osteopathy and Islamic medical practice) are included in the study. Participants in this online survey is voluntary. You may choose not to participate. If you decide to participate in this online survey, you may withdraw at any time. If you decide not to participate in this online survey or if you withdrawal from participating at any time, you will not be penalized.

Procedure:
The procedure involves filling an online surveys will take approximately 15-20 minutes. Your responses will be confidential and we do not collect identifying information such as your name, IC Number, Contact number or IP address. The survey questions will be about to ask from Traditional and Complementary Medicine Practitioners on cyberbullying behaviour.
We will do our best to keep your information confidential. All data is stored in a password protected electronic format. To help protect your confidentiality, the survey will not contain information that will personally identify you. The results of this study will be used for research purposes only.

If you have any questions about the research study, please contact to Dr. Muhammad Shahzad Aslam (aslam.shahzad@xmu.edu.my)

Thank you so much and take a good care.

Best regards
Dr. Muhammad Shahzad Aslam
Xiamen University Malaysia
Version: Questionaire_Aslam_V0.2_22November2020

* 1. I agree that any personal information provided in this online survey can be used for the purposes(s) mentioned in the privacy Notice.

- Agree
- Disagree

* 2. Practice Area

- Traditional Malay Medicine
- Traditional China Medicine
- Traditional India Medicine
- Homeopathy
- Chiropractic
- Osteopathy
- Islamic medical practice
- Other than recognized practice areas by Ministry of health Malaysia

Cyber-bullying among Traditional and Complementary Medicine Practitioners at workplace cross-sectional descriptive study

Email address and their Registration number from Official governing bodies

Your information will kept confidential and only need for verification purpose

* 3. Experience in relevant field (in years)

- 1
- 2
- 3
- 4
- 5
- 5+

* 4. Working Sector

- Government Hospital
- Government Health Clinic
- Private Hospitals
- Private Clinic
- Other

Cyber-bullying among Traditional and Complementary Medicine Practitioners at workplace cross-sectional descriptive study

* 5. Your work performance has been commented upon in negative terms on the Internet

- Strongly agree
- Agree
- Neither agree nor disagree
- Disagree
- Strongly disagree

Cyber-bullying among Traditional and Complementary Medicine Practitioners at workplace cross-sectional descriptive study

* 6. Gossip or rumors about you have been spread on the Internet

- ◯ Strongly agree
- ◯ Agree
- ◯ Neither agree nor disagree
- ◯ Disagree
- ◯ Strongly disagree

Cyber-bullying among Traditional and Complementary Medicine Practitioners at workplace cross-sectional descriptive study

* 7. In what type of community do you work?

- ◯ City or urban community
- ◯ Suburban community
- ◯ Rural community
- ◯ Other (please specify)

Cyber-bullying among Traditional and Complementary Medicine Practitioners at workplace cross-sectional descriptive study

* 8. Threatening personal messages have been sent to you via digital media

- ◯ Strongly agree
- ◯ Agree
- ◯ Neither agree nor disagree
- ◯ Disagree
- ◯ Strongly disagree

Cyber-bullying among Traditional and Complementary Medicine Practitioners at workplace cross-sectional descriptive study

* 9. Persistent criticism of your work or performance has been made against you via digital media

- ◯ Strongly agree
- ◯ Agree
- ◯ Neither agree nor disagree
- ◯ Disagree
- ◯ Strongly disagree

Cyber-bullying among Traditional and Complementary Medicine Practitioners at workplace cross-sectional descriptive study

* 10. Others have commented on the Internet that you should quit your work

- Strongly agree
- Agree
- Neither agree nor disagree
- Disagree
- Strongly disagree

Cyber-bullying among Traditional and Complementary Medicine Practitioners at workplace cross-sectional descriptive study

* 11. Your computer identity has been hijacked

- Strongly agree
- Agree
- Neither agree nor disagree
- Disagree
- Strongly disagree

Cyber-bullying among Traditional and Complementary Medicine Practitioners at workplace cross-sectional descriptive study

* 12. Colleagues have excluded you from the social community online (e.g. Facebook, Twitter)

- Strongly agree
- Agree
- Neither agree nor disagree
- Disagree
- Strongly disagree

Cyber-bullying among Traditional and Complementary Medicine Practitioners at workplace cross-sectional descriptive study

* 13. Rude messages have been sent to you via digital media

- Strongly agree
- Agree
- Neither agree nor disagree
- Disagree
- Strongly disagree

Cyber-bullying among Traditional and Complementary Medicine Practitioners at workplace
cross-sectional descriptive study

* 14. Your mistakes or errors at work are repeatedly commented about in e-mails, text messages, or the like

- ◯ Strongly agree
- ◯ Agree
- ◯ Neither agree nor disagree
- ◯ Disagree
- ◯ Strongly disagree

Cyber-bullying among Traditional and Complementary Medicine Practitioners at workplace
cross-sectional descriptive study

* 15. What is your sex?

- ◯ Male
- ◯ Female

Cyber-bullying among Traditional and Complementary Medicine Practitioners at workplace cross-sectional descriptive study

16. Age group

- ◯ 18-24
- ◯ 25-34
- ◯ 45-54
- ◯ 55-64
- ◯ 65+

Cyber-bullying among Traditional and Complementary Medicine Practitioners at workplace cross-sectional descriptive study

* 17. Viruses have intentionally been sent to your e-mail address

- ◯ Strongly agree
- ◯ Agree
- ◯ Neither agree nor disagree
- ◯ Disagree
- ◯ Strongly disagree

Cyber-bullying among Traditional and Complementary Medicine Practitioners at workplace
cross-sectional descriptive study

* 18. Your supervisor/colleagues are not responding to your e-mails or text messages

- Strongly agree
- Agree
- Neither agree nor disagree
- Disagree
- Strongly disagree

Cyber-bullying among Traditional and Complementary Medicine Practitioners at workplace
cross-sectional descriptive study

* 19. Extracts from your messages have been copied so that the meaning of the original message is distorted

- Strongly agree
- Agree
- Neither agree nor disagree
- Disagree
- Strongly disagree

Cyber-bullying among Traditional and Complementary Medicine Practitioners at workplace cross-sectional descriptive study

* 20. Aggressively worded messages (e.g. capital letters, bold style or multiple exclamation marks) have been sent to you via e-mail, text messages)

- Strongly agree
- Agree
- Neither agree nor disagree
- Disagree
- Strongly disagree

* 21. What is the highest level of education you have completed?

- Diploma
- Professional degree
- Undergraduate
- Masters
- Ph.D.
- Other

Cyber-bullying among Traditional and Complementary Medicine Practitioners at workplace cross-sectional descriptive study

* 22. Which of the following best describes your current relationship status?

- Married
- Widowed
- Divorced
- Separated
- In a domestic partnership or civil union
- Single, but cohabiting with a significant other
- Single, never married

Cyber-bullying among Traditional and Complementary Medicine Practitioners at workplace cross-sectional descriptive study

* 23. Allegations about you have been made on the Internet

- ○ Strongly agree
- ○ Agree
- ○ Neither agree nor disagree
- ○ Disagree
- ○ Strongly disagree

Cyber-bullying among Traditional and Complementary Medicine Practitioners at workplace cross-sectional descriptive study

* 24. Attacks against you as a person, your values or your personal life have been made on digital media

- Strongly agree
- Agree
- Neither agree nor disagree
- Disagree
- Strongly disagree

Cyber-bullying among Traditional and Complementary Medicine Practitioners at workplace cross-sectional descriptive study

* 25. False statements about you have been spread on the Internet

- Strongly agree
- Agree
- Neither agree nor disagree
- Disagree
- Strongly disagree

Cyber-bullying among Traditional and Complementary Medicine Practitioners at workplace cross-sectional descriptive study

* 26. Threatening messages about your friends/your family have been sent to you via digital media

- Strongly agree
- Agree
- Neither agree nor disagree
- Disagree
- Strongly disagree

Cyber-bullying among Traditional and Complementary Medicine Practitioners at workplace cross-sectional descriptive study

* 27. Jokes about you have been spread on the Internet or via e-mail to several recipients

- Strongly agree
- Agree
- Neither agree nor disagree
- Disagree
- Strongly disagree

Cyber-bullying among Traditional and Complementary Medicine Practitioners at workplace cross-sectional descriptive study

* 28. Necessary information has been withheld making your work more difficult (e.g. being excluded from e-mail lists)

- Strongly agree
- Agree
- Neither agree nor disagree
- Disagree
- Strongly disagree

Cyber-bullying among Traditional and Complementary Medicine Practitioners at workplace cross-sectional descriptive study

* 29. Offensive photos/videos of you have been posted on the Internet

- Strongly agree
- Agree
- Neither agree nor disagree
- Disagree
- Strongly disagree

Cyber-bullying among Traditional and Complementary Medicine Practitioners at workplace cross-sectional descriptive study

* 30. Which of the following best describes your current job level?

- Owner/Executive/C-Level
- Senior Management
- Middle Management
- Intermediate
- Entry Level
- Other (please specify)

* 31. City

- Kualalumpur
- Malacca
- Penang
- Kotakinabalu
- Ipoh
- Kuching
- Johor Bahru
- Kuantan
- Alorsetar
- Sandakan
- Kangar
- Kelantan
- Other

* 32. What is your approximate average household income?

- MYR 0-999
- MYR 1,000-2,999
- MYR 3,000-4,999
- MYR 5,000-6,999
- MYR 7,000-8,999
- MYR 9,000-10,999
- MYR 11,000-12,999
- MYR 13,000 and up

* 33. Are you involve in Substance abuse such as Cigarette, Cigar, pipe, tobacco, alcohol, non-prescribed cough syrup or psychoactive pill?

- Yes
- No
